# Annotations

**Published:** 1909-03-20

**Authors:** 


### ANNOTATIONS.

### The Prince and the Medical World.

Even the most casual newspaper reader must have noted the many signs which have lately been given in the most obvious manner by the Prince and Princess of Wales of their deep and continued interest in the progress of the medical sciences and of hospital administration. With the innumerable claims upon their attention in no way abated, the Prince and Princess have yet found time in the course of the past few weeks to encourage the medical profession and the authorities of the metropolitan hospitals by a succession of personal visits and other evidences of practical interest, which, if compared with previous marks of Royal favour during any given space of time, are probably without parallel. The compliment is all the greater when it is remembered that hospital administration and medical practice and education, and all that is allied to both, are at the present time the subjects of an immense amount of criticism not always of the friendliest kind. To mention some of the principal recent activities of the Prince and Princess in the world of medicine shows at once that their range has been considerable. Surprise visits have been made to four of the great London teaching hospitals in rapid sequence; the Royal College of Surgeons has been visited with a double purpose—the hearing of the President's Hunterian oration and the receiving of the Honorary Fellowship by the Prince of Wales, and has since been visited again to inspect some of the specimens in the museum; and, as usual, the committee of the King's Hospital Fund were last week received at Marlborough House by their President on the occasion of the annual meeting, and were addressed by him upon the work of the past year. The presence of His Royal Highness throughout the course of the Inter-Hospital Rugby Football Cup-tie between Guy's and the London Hospitals may seem scarcely to have a bearing on the subject in hand; but the students who thronged the Richmond ground, and welcomed their distinguished visitor with such enthusiasm, may perhaps be allowed to take this notable event in their athletic annals as an intentional sign of Royal interest in the welfare of a much-abused section of hospital workers.

### The Anæsthetics Bill.

The recent discussions between leading anæsthetists in the columns of some of our contemporaries and at the meetings of the Section of the Royal Society of Medicine have revealed much diversity of opinion as to what should and what should not be provided in the contemplated legislation. It seems to have been settled that the suggested and urgently needed prohibition of general anæsthesia by unqualified men should include gas given for dental purposes, a reform which may not prove acceptable to the dental profession; so at least it has been stated in the Press. The point is a very debatable one, whether dentists should thus be excluded from the administration of gas. On the one hand, it seems illogical to allow one particular class of persons not qualified to practise medicine to give one particular general anæsthetic, while absolutely restricting other general anæsthetics to registered medical men; and it is almost certainly a measure conducive to the public safety that general anæsthesia should be declared the province of the medical profession alone. On the other hand, some dentists give gas at least as competently as many medical men; and to cut off from cheap extraction-anæsthesia a large section of the less affluent public is undoubtedly somewhat of a hardship. The Privy Council and the Home Office may be trusted to weigh carefully the pros and cons of the situation. Meanwhile it may be pointed out that Dr. Waldo's proposals, embodied in his annual return to the Court of Common Council, are much more sweeping. He would restrict not merely general but also local anæsthesia to the medical profession. He would have it compulsory to record details of every administration, whether in private or in institutions: to employ in the latter resident anæsthetists whenever possible; and to secure supervision of all junior qualified men by a skilled anæsthetist until the former can procure a certificate of special competence. There are other recommendations also, but those we have quoted are sufficient to show the advanced position which Dr. Waldo takes up; a position with elements of considerable stragetic strength, but as at present framed probably too far in advance of the main body to secure material support.

### An Inoculation Ward.

The Chairman of St. Mary's Hospital, London, speaking on Tuesday last a tthe annual meeting of the Ladies' Help Society in connection with that institution, made an important announcement, which will cause the greatest satisfaction among the friends of the hospital, and throughout the medical world generally. A committee of enlightened and influential gentlemen have just entered into an agreement with the managers of St. Mary's Hospital to provide accommodation and maintenance, in the new Clarence Memorial wing, for 31 patients, who will there receive the new treatment by bacterial inoculation under the care of its discoverer Sir Almroth Wright. This act of far-sighted generosity, has, as Alderman Henry Harben pointed out, a double significance. It will advance a line of scientific work, which has already been turned to much account in the alleviation of disease, and has immense future possibilities; and it will remove the reproach, which for so many years has oppressed the management of St. Mary's Hospital, namely, that of having unoccupied wards in the institution. We have yet to learn the details and principles of the agreement, and the official list of the donors' names; but we can already convey our thanks on behalf of the medical profession to those who are thus endowing an important branch of medical research and extending the scope of medical charity.

